# Unraveling agricultural water pollution despite an ecological policy in the Ayeyarwady Basin

**DOI:** 10.1186/s12889-024-19084-7

**Published:** 2024-06-10

**Authors:** Lazarus Obed Livingstone Banda, Chigonjetso Victoria Banda, Jane Thokozani Banda, Eretia Mwaene, George N. Chidimbah Munthali, Thin Thin Hlaing, Blessings Chiwosi

**Affiliations:** 1 Nalikule College of Education, Kanengo, Lilongwe, Malawi; 2 School of Political Studies and Public Administration, University of Malawi, Zomba, Malawi; 3 Ministry of Education, Directorate of Higher Education, Capital Hill, Lilongwe, Malawi; 4 Beijing Institute of Technology, School of Aerospace Engineering, Zhongguancun, Haidian, Beijing China; 5 School of Economics and Management, Yangtze University, Jingzhou, Hubei China; 6 Huazhong University of Science and Technology, College of Public Administration, Wuhan, China; 7 College of Humanities and Development Studies, China Agriculture University, Haidian, Beijing, China

## Abstract

**Background:**

The Ayeyarwady Basin in Myanmar, a critical economic zone, faces severe ecological degradation due to unsustainable agricultural practices. These practices pose significant threats to human health and marine biodiversity. Environmental threats persist despite the Myanmar government’s efforts to implement biodiversity protection policies. This research explores the limited compliance with environmental protection policies among farmers in the Ayeyarwady Basin and its implications for sustainable agricultural practices and ecological conservation.

**Methods:**

This research employs an exploratory phenomenological approach, utilizing semi-structured, in-depth interviews with government officials and farmers (*N* = 30). The data collected were subjected to thematic analysis using Atlas 23.

**Results:**

Preliminary findings reveal a gap in farmers’ awareness and understanding of these policies, hindered by insufficient financing, poor communication infrastructure, and uncoordinated policy monitoring. These factors and existing unrest contribute to a top-down policy approach that neglects frontline stakeholders. The study suggests the need for clear stakeholder roles, adequate policy financing, and diverse communication strategies to effectively implement environmental policies and protect human and marine life.

**Conclusions:**

Environmental policy shortcomings in Myanmar are attributable to governmental oversight and insufficient stakeholder engagement. To mitigate pollution and safeguard river basin ecosystems, the government must delineate stakeholder responsibilities, allocate appropriate policy funding, and adopt varied communication approaches with farmers.

**Graphical Abstract:**

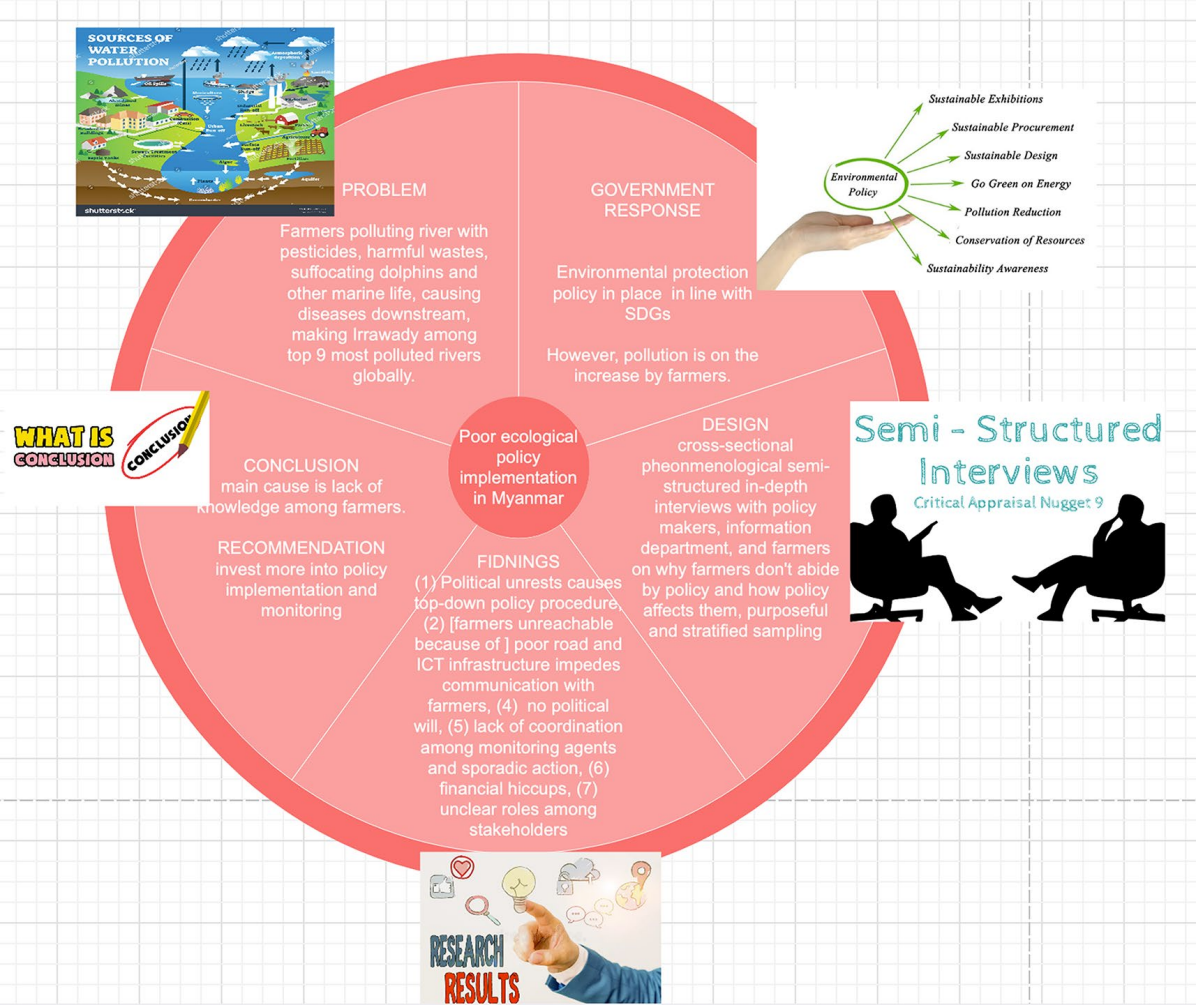

## Introduction

Water pollution is the contamination of water bodies by harmful substances impacting their physical, chemical, and biological properties [1–4]. It significantly impacts ecosystems, human health, and livelihoods, with industrial activities significantly contributing. For instance, a study by Uddin et al. highlights the spatial variability in the distribution of trace metals in groundwater around industrial sites, underscoring the significant impact of industrial activities on water quality [5, 6]. Additionally, terrestrial runoff, including nutrients like nitrogen, veterinary pharmaceuticals, and phosphorus from urbanization, sewage discharge, plastics, pesticides, fertilizers, industrialization, waste disposal, and sewage, severely impacts aquatic and coastal ecosystems, human health, and plant life properties [3, 4, 7]. This deterioration in water quality endangers habitats and the coastal communities dependent on them [2–4, 7].

The regional challenges are exemplified in Cambodia, where inadequate wastewater treatment infrastructure, insufficient regulations and enforcement, agricultural runoff containing pesticides and fertilizers, industrial waste disposal, population growth, and urbanization have resulted in significant water pollution [8, 9]. This pollution, combined with the export of sediments from agricultural areas, has degraded water quality and threatens aquatic ecosystems downstream [10]. These challenges are further compounded by the effects of climate change, such as rising sea levels and extreme weather events, which contaminate land and water resources, impacting livelihoods and food security [11]. The increase in foreign direct investment has also impacted the environment [10]. In Thailand, particularly in areas such as Songkhla Lake, unsuitable practices for rubber plantations, palm oil, and deforestation have led to increased soil erosion and sedimentation in the lake [12]. Similarly, in Ukhiya, Bangladesh, significant deforestation and changes in land use contribute to water quality degradation [13].

Marine ecosystems, crucial for global ecology [7], face increasing pollution threats, mainly from land-based human activities [7, 14]. Research, including OECD findings, identifies unprocessed waste discharge from industrial and agricultural activities as a principal pollution source. Notably, agriculture’s role in aquatic pollution in developing nations is comparatively lesser than that of urban and industrial contributions. Detailed case studies, such as the one on groundwater contamination near industrial areas [5], the impact of land use on river water quality in Bangladesh [15, 16], and irrigation water contamination and health risks in Addis Ababa [16], highlight similarities and differences with the Ayeyarwady Basin, and the utility of advanced WQI models [5].

In East Asia, agricultural non-point source pollution is a primary environmental concern, particularly in China. The primary contributors to water pollution include increased use of fertilizers and inadequate livestock waste disposal, impacting water quality alongside industrial and domestic activities [17]. The prevalent use of intensive farm chemicals and inadequate waste management practices compound the water pollution issue [18, 19]. This nexus highlights the complex interaction between agricultural practices and aquatic contamination. Additionally, escalating consumerism, characterized by increased demand for diverse products and services, augments production and waste [4], contributing to substantial environmental stress and further compounded by climate change effects.

Water pollution indirectly influences climate change by altering the chemical composition of water bodies, affecting their ability to absorb and reflect sunlight and potentially influencing local weather patterns and temperatures [3]. Moreover, water pollution damages ecosystems like wetlands, oceans, and vital carbon sinks, reducing their ability to absorb atmospheric carbon dioxide and accelerating climate change [20]. Pollutants from landfill leachate, agricultural runoff, and sewer system infiltration significantly contribute to greenhouse gas emissions [21–24], further complicating environmental challenges.

Recognizing the importance of healthy marine environments for human communities, the United Nations Sustainable Development Goals highlight the need to protect marine resources, emphasizing their vital role in global health and the economy [11, 15]. This connection underscores the importance of sustainable farming practices to protect water quality [2, 25]. Mitigating water pollution is crucial for the ecosystem, human health, and addressing climate change impacts [7, 26]. Heightening awareness to mitigate the adverse impact of water pollution is timely [7].

In the Ayeyarwady region of Myanmar, farmers significantly exacerbate water pollution through excessive water use in irrigation, untreated wastewater for irrigation purposes, overuse of fertilizers and pesticides, and pollution of irrigation water drains and valley estuaries [14, 27]. Myanmar’s economy faces many internal and external problems [14], with around 40% of the population living in poverty since 2022. The Ayeyarwady region is known as the region’s rice bowl due to its large-scale agriculture [28]. A study by Gani et al. assessed the impact of land use and land cover on river water quality using water quality index models and remote sensing techniques, highlighting significant correlations between land use types and water quality indicators [15].

Climatically, Ayeyarwady experiences a tropical monsoon climate with considerable rainfall throughout most of the year [29]. However, environmental risks such as storms, floods, high temperatures, droughts, and rising sea levels threaten the basin’s agricultural productivity, directly impacting Myanmar’s future food security [19]. Despite extensive climate change management and adaptation research, agricultural water pollution in Myanmar remains high [30]. This pollution, involving plastics, fertilizers, and pesticides, has made Ayeyarwady the ninth most polluted globally [31]. The DeRisk SE Asia project, a comprehensive initiative for climate risk management in Southeast Asia, highlights the multidisciplinary approach required to address these challenges [32].

However, a significant gap remains in farmers’ understanding and application of these regulations and knowledge, underscoring the need for enhanced awareness and integration of ecological practices in farming. Investigating farmers’ awareness, knowledge about policy, and perceptions concerning safeguarding the region from degradation to ensure sustainable agricultural economic growth is essential. The study aims to investigate the influence of farmers’ comprehension of environmental policies on their agricultural practices in Myanmar, conducting an in-depth analysis of farmers’ awareness and interpretation of governmental ecological regulations. Understanding how this knowledge shapes farmers’ practices in mitigating environmental degradation and contributing to sustainable agricultural economic growth in the region is crucial.

The significance of this study lies in its novel assessment of environmental regulation awareness among the farming community of the Ayeyarwady River Basin. It offers critical insights into the interplay between ecological regulations and economic development, particularly under global climate change. Such insights are vital for developing policies that effectively balance economic resilience, ecosystem protection, and sustainable growth. Additionally, the research contributes to the broader discourse on Sustainable Development Goals by integrating diverse perspectives from farmers, public officials, and experts. This comprehensive approach is pivotal in informing policy decisions that reconcile agricultural economic growth with biodiversity preservation, thereby shaping Myanmar’s greener and more equitable future.

The rest of this paper is sequentially organized into distinct sections, beginning with the research methodology, research findings, and discussion and culminating in the conclusion.

## Design and methodology

This study adopted a qualitative approach. We decided to use this approach, mindful that stakeholders typically have different lived experiences, and each scenario would need in-depth analysis to understand the underlying data. This study was phenomenological in which stakeholders shared their experiences with the prevalent environmental policy and agricultural production under such policy.

Qualitative research constitutes a methodological approach fundamentally oriented towards elucidating subjective phenomena, encompassing feelings, ideas, and experiential dimensions [33]. The cardinal aim of data acquisition within this paradigm is eliciting insights conducive to generating hypotheses amenable to empirical testing. Predominantly narrative in its mode of data representation, qualitative research is instrumental during the preliminary stages of investigative endeavors, facilitating the discernment of emergent patterns and novel interpretative frameworks [34]. As a distinct research methodology, it is characterized by its focus on non-quantitative data. It eschews statistical methods in favor of semi-structured or unstructured methodologies, enabling a more profound exploration of the research subject matter [33].

### The geographical setting of this study

**Fig. 1:**
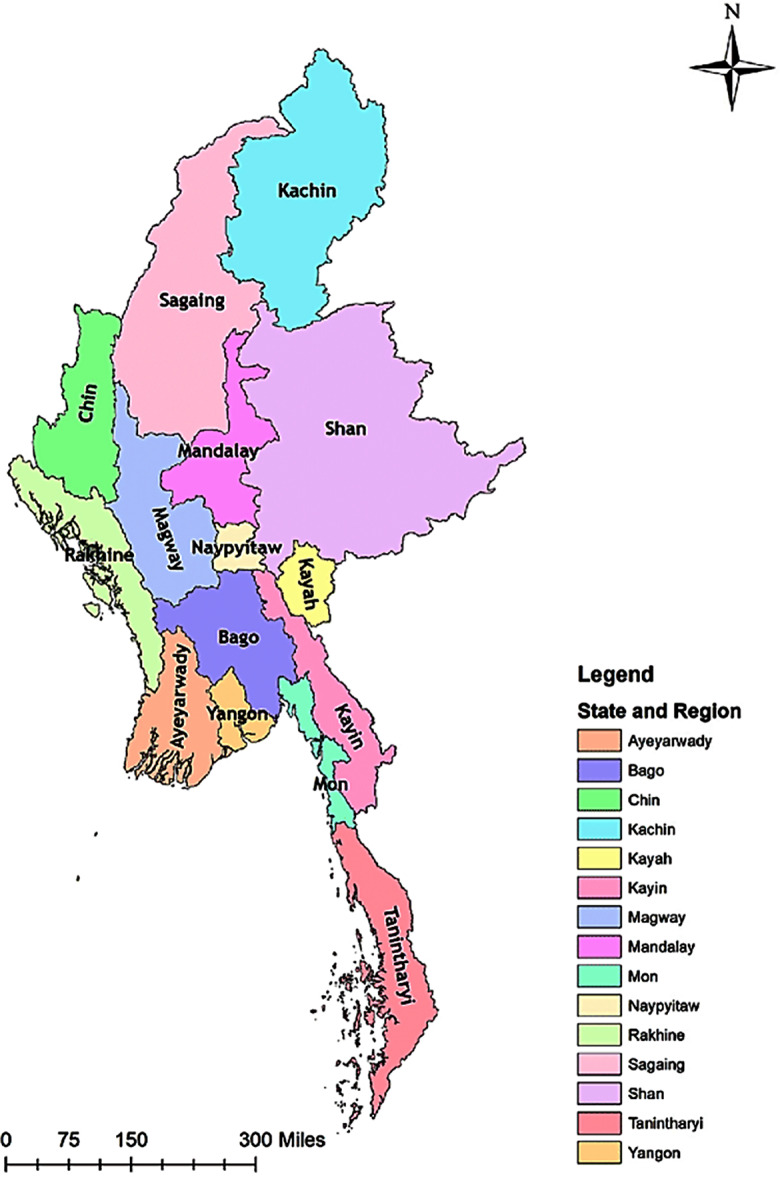
the map of Myanmar showing these states and regions. Source:https://www.researchgate.net/figure/Map-of-Myanmar-showing-states-and-regions_fig1_339105755

To understand the concept of regional development in the case of Myanmar, the study will begin with an exposition of the country’s geographical and political setting. Figure 1 is Myanmar’s map showing these states and regions.

Two main geographical settings are of interest in this study. These are the Ayeyarwady Region and the Naypyidaw Union Territory. The Delta Region is another name for the Ayeyarwady Region in the southern part of the continent. The colossal estuary of the Irrawaddy (Ayeyarwady), the principal river of Myanmar, is the coastal region, roughly half the size of Ireland, and is situated between the Bay of Bengal and the Gulf of Martaban.

Formerly, Naypyidaw Union Territory belonged to the Mandalay Division. The territory is home to the Government of Myanmar’s seat and the nation’s recently established artificial administrative capital. The Union Parliament, the Presidential Palace, the Supreme Court, and multiple golf courses are all in Nay Pyi Taw. As the capital, this was likely to have the highest number of administrators, policymakers, and other top-ranking public officers in the country. Therefore, we purposefully selected this union territory for respondents in the category of public officers.

Climate change and global warming pose significant threats to the agricultural sector in Ayeyarwady. If farmers are not geared towards combating or mitigating their effects, sustainable agrarian activities will not be possible soon. Therefore, farmers must be well-versed in sustainable development strategies to boost agriculture and ensure a sustainable industry.

### Sampling and sample size

Naypyidaw Union Territory, the capital city of Myanmar, was purposefully selected as the focal point of our research. The rationale behind this choice was twofold. Firstly, Naypyidaw is the epicenter of policy development, making it an ideal location for accessing a concentrated pool of knowledgeable and influential individuals. Secondly, the city is the administrative seat where high-ranking officials, including policymakers and officers from the Ministry of Information and Agriculture, are predominantly stationed. Naypyidaw’s geographical and organizational centrality facilitated access to critical informants actively engaged in policy formulation and implementation. The Ayeyarwady region has more smallholders than large-scale farmers [27, 35]. To boost the sample size, we stratified them and officials into groups with minimal variability within each group and maximal variability across groups to enhance estimate precision [36].

We sampled farmers using stratified sampling. From each stratum, we randomly sampled members on -first-interviewed basis. This means that any farmer in each category was available first was requested to participate. We used purposeful sampling [37] to identify participants instrumental in providing adequate data. A stratified sampling methodology was employed to select individual officers for participation in the current investigation. Given the multidisciplinary nature of policy development, implementation, and monitoring, it was essential to ensure that diverse viewpoints were adequately captured. This technique necessitated the division of the target population into distinct strata, each reflecting a key characteristic relevant to the research inquiry, as the officers from each ministry possessed unique insights and expertise relevant to their specific domains: at the policy echelon, specifically from the Ministry of Information—the Ministry overseeing natural resources and environmental stewardship and implementation from major and smallholder farmers.

Therefore, we selected constituent elements from our population after systematically categorizing them into homogeneous, non-overlapping segments. Within each stratum, elements were selected via a simple random sampling process, ensuring each segment’s representation within the overall sample framework [38]. There was a need to ensure representativeness across distinct strata, delineated by their respective roles in policy formulation, dissemination, and implementation [38]. The design facilitated a higher degree of precision in the estimation process, as it controls for variability within distinct subgroups of the population. Following the selection process, the samples derived from each stratum were amalgamated to form a consolidated sample representative of the entire population. This amalgamation was instrumental in providing a microcosmic reflection of the population, thus allowing for more accurate inferences from the data collected [21, 39]. The design ensured that the insights garnered would accurately reflect the perspectives of those involved in orchestrating national policies.

The stratification was further refined to align with the ministries related to the study’s aims. Officials from the Ministry of Information were explicitly included due to the critical role of effective communication in policy dissemination—an area for which this ministry bears the primary responsibility. Therefore, the rationale for adopting this sampling strategy was rooted in its capacity to facilitate a comprehensive understanding of the inter-ministerial dynamics and the intricacies of policy dissemination, ensuring that the resultant data encapsulates the multifaceted nature of policy execution.

Our research methodology encountered specific challenges associated with purposeful and stratified sampling. Purposeful sampling presented issues of subjectivity, as our biases in selecting participants could influence the sample and affect the generalizability of our findings. Additionally, due to its subjective nature, the sample’s representativeness was a concern, as was the study’s replicability. To address these challenges, we established clear and transparent participant selection criteria, mitigating the potential for bias. We also triangulated data sources to enhance the representativeness and validity of the findings and meticulously documented the sampling process to improve the study’s replicability.

In the case of stratified sampling, the complexity of forming appropriate strata that accurately represented key population characteristics was a significant challenge. Allocating the correct sample size to each stratum to ensure statistical efficiency posed another difficulty, as did the requirement for detailed knowledge of the population for effective stratification. We conducted a thorough preliminary population analysis to overcome these obstacles, which aided in effective strata formation. We utilized proportional or optimal allocation methods to determine appropriate sample sizes for each stratum. Furthermore, we leveraged existing data sources and conducted preliminary research to gather population insights for effective stratification. These measures significantly enhanced the quality and relevance of our research findings.

### Data collection

A semi-structured, in-depth, face-to-face interview was chosen as an appropriate approach for this research. For this study, we collected data from March 1st and June 29th, 2023, through semi-structured offline interviews with 6 public officers, 4 mega farmers, and 20 smallholder farmers to deeply understand their level of awareness about the existence and understanding of the prevalent policy. As opposed to a mega farmer, in Myanmar, the formal delineation of a smallholder farmer is established as an individual managing an agricultural expanse not exceeding 3 acres (approximately 1.2 hectares) of cultivable land. This definition underscores the variability inherent in the criteria for classifying smallholder agriculture, which may fluctuate based on a range of agronomic conditions and the specificities of different geographical regions [40].

Before deployment, the questionnaire guide was rigorously evaluated to confirm its face and content validity, utilizing the expertise of three specialists. A pilot test with five key stakeholders was conducted to refine any inconsistencies. Data collection was carried out by research assistants fluent in both Myanmar and English, ensuring linguistic comprehensiveness. The semi-structured interviews utilized a bilingual questionnaire to facilitate participant comprehension. A dual-method approach was adopted for accurate data capture: audio recordings using smartphone applications concurrently with note-taking on an iPad, conditional on interviewees’ informed consent.

After the data collection phase, bilingual experts proficient in the local language and English undertook a meticulous transcription process. This involved converting voice recordings from the local language into written text and carefully translating the material into English. Ensuring the accuracy and fidelity of these translations was paramount in preserving the original context and nuances of the responses.

Once the transcription and translation processes were completed, we imported the data into Atlas 23.2.1. This advanced software facilitated an expedited, comprehensive, and detailed analysis of the transcripts [41]. Central to our analytical approach was the execution of sentiment analysis within the same version of Atlas. This phase was predominantly automated, capitalizing on the artificial intelligence capabilities of the platform. This automation allowed efficient and systematic initial data coding, identifying key patterns and sentiments.

However, we recognized the limitations of an exclusively automated analysis, particularly its ability to fully capture human communication’s subtleties. Besides, there is a growing need for explainable AI as AI-powered analytics become more widespread, and the decision-making process of AI systems can be opaque, leading to difficulties in understanding and trusting the results produced by AI algorithms [41].

Consequently, we supplemented this with a manual code review, enabling us to delve deeper into the context and finer aspects of the data, providing a richer and more comprehensive analysis. This review phase was critical in integrating a more nuanced understanding of human gestural communication, which often eludes purely algorithmic interpretation. By combining the technological efficiency of Atlas’ AI-driven analysis with the depth of human interpretative skill, we achieved a balanced and synergistic analytical approach. This methodology allowed us to leverage the strengths of both automated and manual analyses, ensuring a thorough, insightful, and contextually sensitive interpretation of the data.

Utilizing the advanced capabilities of an artificial intelligence engine, we initially generated a comprehensive set of 271 codes from the interview transcripts. To ensure accuracy and relevance, each code was meticulously verified by manually examining the transcripts. This exhaustive process enabled us to distill the initial set into a more focused collection of 16 memos corresponding to the most frequently applied codes. Among the government officials, our analysis identified seven predominant co-occurring themes: long-term economic costs, compliance costs, sustainable development, perspectives of farmers, environmental degradation, agricultural production, and extension services.

During the analytical phase, we identified and consistently applied the 16 primary codes across the dataset, mainly focusing on the responses from both government officials and academic participants. This coding process was integral to our qualitative data analysis methodology, facilitating the systematic categorization and interpretation of the complex dataset.

The employment of sophisticated graphical representations marked the culmination of our analytical phase to visualize the qualitative interview data. These visual tools were instrumental in elucidating the intricate interplay of themes within the dataset. We utilized network maps to delineate the interconnections between various codes, thereby mapping the complex relationships and linkages among diverse themes. Force-directed graphs were employed to illuminate the relational dynamics among these themes, providing a deeper understanding of how different concepts were interrelated within the context of our study.

Moreover, we generated word clouds to accentuate the most frequently occurring terms within the interviews, visually representing the predominant concepts and ideas. Additionally, tree maps were utilized to provide a hierarchical view of the data structure, enabling us to discern the organization and relative significance of various themes and subthemes within the dataset. These graphical representations were pivotal in facilitating a nuanced and comprehensive understanding of the dataset’s thematic complexity. They enabled us to distill and present the qualitative data intelligibly and thoughtfully, significantly enhancing our interpretative analysis.

We opted for in-depth interviews as they facilitate the creation of a platform for mutual idea exchange between the researcher and the participant. This interactive format allows researchers to gain a profound understanding of participants’ perspectives and insights [42], while participants can benefit from the researcher’s expertise in the specific field [43]. In-depth semi-structured interviews are particularly effective in qualitative research for capturing the intricacies of participants’ experiences and viewpoints [43]. This methodology enables researchers to delve into and clarify participants’ responses, establish a rapport fostering trust and openness, and customize the interview to align with specific research objectives [42]. It provides invaluable insights and a deeper comprehension of the studied phenomenon.

Moreover, in-depth interviews are adept at identifying trends and patterns within the data. By examining the responses of multiple participants, researchers can discern common themes and tendencies, which enrich the findings and suggest avenues for future research and hypothesis generation [43].

One of the critical advantages of interviews is the ability to ask follow-up questions or seek clarification when responses are unclear or ambiguous, ensuring data accuracy and completeness. This versatility or adaptability allows researchers to explore unforeseen topics during the interview, leading to a more exhaustive understanding of the research subject [42, 43].

However, the use of interviews comes with its set of challenges. Recognizing these, we complemented our qualitative approach with quantitative data. In-depth semi-structured interviews can be demanding regarding resources and time and are prone to interviewer and social desirability biases. The interviewer’s communication style may affect the participants’ openness in sharing their experiences and viewpoints. We maintained a manageable sample size of 30 interviewees to mitigate these challenges.

Interviewer bias, where the interviewer’s preconceptions influence question framing or interpretation of responses, can lead to skewed data [42]. Additionally, social desirability bias may prompt participants to offer responses they perceive as socially acceptable rather than genuine thoughts and feelings. We addressed these issues by ensuring confidentiality and anonymity, emphasizing that there were no right or wrong answers to encourage candid and accurate responses. Moreover, the interviewer’s communication style may affect participants’ willingness to share their experiences or perspectives [43]. The interviewer’s communication style can affect participants’ desire to share their experiences or perspectives. For instance, an inquisitor perceived as judgmental or dismissive may discourage participants from expressing their true thoughts and emotions. For this reason, we ensured that the interviewers were well-versed in such challenges and always conscious of the same. These were professional data collectors.

### The interview guide, duration, and procedure

This investigation meticulously crafted semi-structured and open-ended inquiries, eschewing leading questions and dichotomous (yes/no) queries, except when strategically intended. The overarching design of the study guide was predicated upon ensuring the consistency and lucidity of the questions, including prompts and subsequent follow-up inquiries.

Consequently, while the anticipated duration for each interview was preliminarily estimated to average 45 min, the actual engagement with interviewees frequently extended beyond this temporal boundary, notwithstanding the brevity of the initial question set. Accordingly, participants were apprised at the outset of each interview session that the process would encompass a minimum of 45 min and not exceed one hour. Prospective participants unable to allocate a minimum of 45 min were precluded from participation to circumvent the precipitous navigation through inquiries, thereby safeguarding against the truncation of comprehensive information requisite for exhaustively addressing the subject matter.

Each interview session was initiated with the formal presentation of official introductory documents sanctioned by the school department overseeing this study. Given the human-centric nature of this research, the interview process encompassed a detailed exposition of the ethical standards incumbent upon the data collectors. Central to this ethical framework was adherence to the Declaration of Helsinki, an eminent compendium of ethical principles about human experimentation, initially formulated in 1964 by the World Medical Association and considered a cornerstone in human research ethics. The research protocol scrupulously delineated ethical considerations, affirming its alignment with the tenets of the Declaration. Following Article 30 of the Declaration, the ethical evaluation comprehensively encompassed considerations of the participants’ interests after the study’s conclusion.

Research questions.

To achieve the objective of this study, the investigation was guided by the following research questions:

1. To what extent do the farmers know about the existence of the policy that regulates farming?
2. How do farmers understand and interpret this policy in their daily agricultural activities?
3. How do farmers understand the relationship among agriculture, environmental protection, and economic development?

## Results

The sample comprised both male and female respondents. To have a more representative sample, we tried to balance gender representation where possible. 50% of the participants were male and 50% female. 4 were mega farmers, while 20 were smallholder farmers (as these are most farmers in Myanmar, mainly residing in the rural farmlands). 2 officials were from the Ministry responsible for Environment and Natural Resources, 2 from the Ministry of Agriculture, and 2 were academicians. Most participants were within the age range of 32 to 35, while the least number of participants were between 20 and 23. Table 1 shows the demographic characteristics of the respondents.

**Table 1:** Demographic characteristics of the respondents

Serial	Designation	Male	Female	Total		Age range	Freq
1	Mega Farmers	2	2	4		20–23	6.7%
2	Small scale farmers	9	11	20		24–27	20%
3	Environment ministry officials	1	1	2		28–31	23.3%
4	Agriculture ministry officials	1	1	2		32–35	36.7%
5	Academicians	2	0	2		36+	13.3%
6	N			30			

The frequency in the last column in Table 1 is not by stratum (not by designation or occupational roles) but by age range; for instance, 13.3% of participants were at least 36 years old.

We carried out data analysis in two distinct yet similar phases. One phase was with data from public officers. The other phase was with farmers. We decided to separate the analysis to identify discrepancies between these distinct groups of participants. The codes generated from responses from policymakers and administrators reveal an emphasis on advocacy, lobbying, and increased policy awareness. They alluded to the lack of understanding of the policy content, the link between environmental conservation policy and sustainable agricultural investment, outputs, and economic development. These participants say that environment, agriculture, and economic growth are inextricably intertwined. A failure in one may lead to a loss in the others.

From these codes, we developed four themes in line with the study objectives and prevalent literature:

(1) Stakeholders’ awareness of the presence of the policies;

(2) Stakeholders’ understanding of the policy;

(3) Stakeholders’ understanding of the relationship among environmental policy;

(4) Agricultural economic development and sustainability of agriculture and regional development.

### Theme 1 stakeholders’ awareness of the presence of the policies

One of the public servants believes that less than 50% of farmers are aware of the public policy regarding safeguarding natural resources in the country. She highlights the need for more advocacy and education on policy implementation:

“Less than 50% of the farmers might understand these policies. Most farmers are unaware, leading to a high level of misunderstanding about the government’s intention behind introducing this policy.” (A public officer)

A peasant farmer acknowledges having heard about public policy related to ecological conservation. However, the participant also explained that she did not understand why these laws should significantly affect the agricultural industry. The farmer indicates her level of lack of familiarity with the policy in the following quotation:

I am just aware of some rules but don’t understand them. So in short I don’t know the details about them. (A female farmer).

There will be many laws related to environmental protection. Nevertheless, I don’t know the details. I know that there is no other way to use the land. I’m unaware of the details of these policies. I lack that knowledge (a male farmer).

Asked about the same, another farmer also expressed a lack of awareness about the policy.

I am unaware of environmental policy.

In contrast to the perspective offered by the female farmer, a male counterpart articulated a lack of awareness on the subject. He echoed a call for increased educational efforts, a common sentiment among farmers. The subsequent excerpts are attributed to this male farmer: I don’t know about those rules. Maybe they have. I have no idea about the existence of any policy.

Besides his unawareness, this man also indicated that he believed many more farmers may be in his shoes. Consistent with one of the public officer’s views, this farmer opined that much as the policies may exist, few farmers might be aware of the same. The quotation below explains his views:

I think very few people know whether those policies exist. However, We need to learn more about that policy. It will help us protect natural resources more.

Although unfamiliar with the existing policies, the male farmer recognized the potential benefits of policies focused on preserving ecological artifacts and resources for sustainable agricultural practices and their contribution to economic development. He emphasized the crucial role of public policy in maintaining productive agriculture and fostering sustainable development. His views are captured in the following statement:

Nowadays, farmers are using too much fertilizer, which has side effects on the soil and will damage natural resources. Knowledge and implementation of such policies will be helpful.

Sorry, the truth is that many of the farmers don’t understand very well.

### Theme 2 stakeholders’ understanding of the policy

It is one thing to know about the presence of a policy yet another to understand what it says. A top-ranking public servant echoed the same concerns about policy awareness among farmers. Regarding the farmers’ awareness of public policies regarding environmental protection, the respondent noted disparities in awareness and the importance of extension services, traditional knowledge, and local initiatives.

The level of awareness among farmers varies widely depending on their location, education, and access to information. However, wide awareness gaps are necessitating more engagement with the farmers. More farmers don’t know the truth about the importance of this policy. Because of inadequate awareness, they feel like the policy oppresses their progress.

Some farmers acknowledged that they knew about the policies’ existence but did not understand them. The following quotation indicates their sentiments.

I don’t understand very well. We are just doing agriculture in the place that they allow. We don’t know why we are not permitted on other sites. We obey because they force us not to use different pieces of land. (female farmer).

We don’t understand the policy. We don’t even know it. No one tells us about it. There is no awareness. No advocacy coupled with appropriate information. We should know more about the public policy regarding safeguarding, protecting, and enhancing natural resources. I think it is a weakness in our country. (female farmer).

Another farmer lamented likewise. Much as he acknowledged the importance of using regulations, there is no clarity about these rules:

From Myanmar’s development and poverty reduction point of view, the agriculture sector is of central importance. However, the problem is that we are prevented from using some resources for more food production. We don’t understand why.

The public officers identified various issues influencing how agricultural economic development contributes to sustainable economic growth. These include advocacy, lobbying, and garnering public support, all crucial for advancing relevant agendas. Awareness and education emerged as key themes, emphasizing addressing misunderstandings, bridging knowledge gaps, and overcoming complete unawareness. Ensuring access to information was highlighted as a vital factor. The officers emphasized the critical importance of balancing environmental conservation with agricultural productivity. They highlighted the necessity of comprehensive civic education and the active involvement of farmers in policy formulation as essential for creating policies that integrate agricultural economic development with sustainable objectives. A significant challenge identified is the widespread misunderstanding of existing laws. Misinformation can be more detrimental than ignorance. Recognizing diverse stakeholder perspectives, respondents underscored the need to balance agriculture and conservation, economic opportunities, climate resilience, resource scarcity, and socioeconomic impacts:

Some farmers have also adopted environmentally beneficial practices like conservation tillage and improved manure storage. However, others don’t understand their importance. They look at things from their perspective alone. Such farmers misunderstand the links between agriculture, the environment, and sustainable development.

### Theme 3 stakeholders’ understanding of the importance of the policy

When asked about the impact of abolishing environmental policy on agriculture’s economic development, she expressed concerns that it would be detrimental.

Many farmers misunderstand. And this misunderstanding leads to adverse implementation challenges. If policy implementation is challenged, sustainable agriculture is impossible in the long run. Thus, sustainable economic development is also jeopardized. It is at stake (an officer).

I don’t understand the relationship between environmental policy, agriculture production, and economic development, but I think environmental protection, agriculture, and the economy are related (a farmer).

On the same, another respondent had the following to say:

“Therefore, as of now, I think farmers are still failing to balance between the policy demands and the agricultural practices that would contribute to economic development in this region. This is because of misunderstanding and low awareness (an officer).

### Theme 4 stakeholders’ understanding of the relationship among environmental policy, agricultural economic development, and sustainability of agriculture and regional development

Regarding the relationship between environmental policy, agriculture production, and economic development in her region, she states that they are interconnected but complex for average farmers to understand. Some farmers view government policies as oppressive to their agricultural economic activities. However, she emphasizes the importance of collaboration between farmers, policymakers, and the agro-food value chain to address environmental challenges.

Bad agriculture practices by the farmers harm the environment. A poor environment, for example, land undergoing significant soil erosion, means too much agricultural input for the farmers to realize some output. That is negative on the regional economy. Cultivating river banks leads to erosion, siltation, and floods, which are detrimental to economic growth.

Another farmer also acknowledged the importance of having policies despite not understanding them:

High yields mean more economic development. But they prevent us from using some land. So, how can we have increased yields? Abolishing the rules ultimately will adversely or negatively affect the country’s natural resources and economic development (farmer).

Environmental protection, agriculture, and the economy are related to each other. Many farmers prioritize agriculture more than preserving the environment. Because it is essential for their business, it’s best to use and keep all of them balanced (farmer).

Of striking importance was a revelation from one public officer lamenting monitoring service and policy implementation:

Numerous organizations are responsible for water safety, but each does as they wish without coordination. Sometimes, it is due to a lack of resources, while others fail due to a lack of interest. Things do not go well where they have vested interests (officer).

Asked whether abolishing the policies would be helpful for the farmers, the consensus was that such regulations were significant to agricultural production and should not be repealed.

I think abolishing any environmental preservation policy will harm agriculture and economic development (farmer).

Abolishing the policy is not good. Maybe the government should do more awareness campaigns to make us understand the procedure. It should have some rules and be shared with people (farmer).

Network analysis indicated intricate relationships among these aspects. When asked about the relationship between environmental policy, agriculture production, and economic development in Myanmar, another senior public officer described it as complex and emphasized the importance of sustainable agricultural practices and responsible resource management for long-term economic growth. They stressed the need to increase awareness among farmers and provide support for policy implementation.

Environmental policy, agriculture production, and economic development are inextricably interrelated, so many average farmers misunderstand it and think the policy opposes their economic activities. However, farmers, policymakers, and the agro-food value chain need more effort and cooperation to address long-standing environmental challenges. I’m privileged to understand the relationship, but many farmers don’t. (officer).

Additionally, the negative perception of a country’s environmental practices can deter foreign investment and tourism, further affecting economic growth. The respondent opined that abolishing the policy would adversely impact sustainable agriculture. Balancing agricultural practices with environmental protection is essential to ensure a sustainable and prosperous future.

### Theme 5 factors compromising policy unawareness levels, significance, and its connection with other sustainable development aspects

The preceding sections have tackled the levels of awareness and understanding among officers and farmers. In this section, we present the participants’ views about factors contributing to low-level awareness and knowledge about extant policy, its implications, and relevance.

To begin with, officers attributed low levels of awareness and complete unawareness about the policy, its importance, and its relationship with other sustainable development factors in Myanmar in some cases. It was apparent that there was a higher level of awareness among the officials than the farmers. This was due to several factors. One policymaker suggested a more integrated approach to effective policy implementation. He suggested that:

The involvement of all stakeholders should begin at the earliest stage of policy formulation. Those at the grassroots need to be included in the situational analysis, policy drafting, dissemination, and implementation. (Policy maker).

According to one public officer at Yangon University, the knowledge gap is loathsome and detrimental. The respondent believes government rules and policies to protect the environment while promoting economic development through agriculture are essential. However, farmers lack awareness about these policies because the Government is not serious about advocacy and awareness campaigns. Consistent with Gani’s [15] and Quader’s [13] ideas, the participant recommends that the Government do a better job of educating and informing the public since environmental policies can significantly impact agriculture production by setting rules for land use, water management, pesticide use, and farming practices. Without the ecological policy, agriculture could harm the environment and negatively impact economic growth. The following quotation highlights that the respondent sees a positive relationship between environmental policy, agriculture production, and economic development.

“The government is weak in educating the public about environmental protection. We haven’t been that vigilant with advocacy” (Officer).

Another public officer from the Ministry of Information echoes these sentiments. She stresses that one reason that weakens information dissemination among farmers is the typological characteristics of our country. She expresses that some farmers are beyond reach due to the terrain.

Reaching out to some remote farmers in the countryside is challenging. This is a problem for us to reach those places for advocacy and awareness and for the farmers to transport their produce to better markets. The government is making all efforts within its abilities to address the challenge. (official).

You barely see cable or any other form of television in some places. Farmers are alienated from the mass information world. They neither use television nor mobile technologies. (official)

Some farmers reiterated the communication concerns raised by the previous respondents above. One lady complained of inaccessibility of information, which compounds her challenges:

If the policies you discuss are disseminated through television, how do you expect this rural area to access that information? Mobile technologies are not as robust as they may be used in towns. All I know about mobile technologies is that they can be used to pick up a call or to listen to a voice message. You need to reach out to us at the most convenient time. Otherwise, we remain entirely unaware of such policies. (farmer)

Some officers attributed the challenges of information dissemination to a lack of appropriate communication infrastructure. Subsequently, they had the following to say:

The Government is facing financial challenges. Through the Ministries of Communication and the Ministry of Transport and Public Works, the Government plans to improve the roads in rural areas and install a backbone for ICTs. (officer).

Two politicians among the participants alluded to the social unrest as an impediment to policy implementation:

Civil and political unrest have severely disrupted our efforts in environmental policy implementation. They create an environment of uncertainty, and fear hampers our ability to engage effectively with local communities. Environmental issues often take a back seat when people are focused on immediate safety and political concerns. Additionally, these unrests disrupt communication channels and logistical plans, making disseminating information about environmental policies and practices challenging. The need for a peaceful and stable environment is paramount to ensure that our efforts in protecting the ecosystem are not in vain (Officer 3).

The ongoing civil and political unrest has profoundly impacted our policy implementation strategies. In some cases, they have necessitated a shift to a more top-down approach, which, unfortunately, can overlook the input and participation of grassroots stakeholders. These unrests also strain our resources, as we must allocate efforts towards maintaining stability and addressing immediate concerns, which can divert attention from long-term environmental goals. We are continuously working to improve our communication strategies and to find ways to better involve local communities in policy discussions despite these challenging circumstances (Officer 3).

Despite the low levels of awareness, respondents in public offices believe that abolishing the environmental policy would harm economic development in the agricultural sector.

The absence of policy could hurt economic growth. This is because these are closely linked. The policy protects natural resources on which agriculture relies. Irresponsible agriculture harms them; in the long run, agricultural activities cannot run effectively anymore to produce a sustainable economy (officer) .

One official vehemently indicated that the government strategy:

There is a significant lack of clear strategic objectives or robust success criteria. Those responsible for awareness lack clarity on processes and methods of carrying out the responsibilities (officer).

Echoing these sentiments, other officers indicated that lack of synergy was a great challenge, coupled with inadequacy of resources:

We’re facing significant hurdles in disseminating information due to poor cable TV connectivity in rural areas. This makes it hard to reach some farmers with critical updates on environmental policies (Information Officer 1).

Indeed, another major issue is resource scarcity. We lack sufficient resources to conduct extensive awareness campaigns, which limits our ability to educate farmers about sustainable agricultural practices; sometimes, the meager resources are used on redundant tasks due to a lack of proper coordination among stakeholders (Officer 2).

There’s often confusion about roles in policy implementation. Sometimes, we’re not clear on who is responsible for what. This role confusion leads to inefficiencies and reduces the overall effectiveness of our environmental initiatives (Officer 1).

The respondent, a Senior Staff Officer, discussed the government rules and policies to protect the environment and promote economic development through agriculture. The officer highlighted that these policies are subject to change, and the country has faced deforestation and environmental sustainability challenges. The respondent emphasized the importance of consulting official government sources for up-to-date information.

Regarding the impact of abolishing environmental policies on agricultural economic development, the respondent highlighted potential consequences such as environmental degradation, reduced agricultural productivity, loss of biodiversity, adverse health impacts, economic vulnerability, and social unrest. They emphasized that sustainable agriculture, promoted by environmental policies, is crucial for food security, natural resource conservation, and resilient economic development. They stressed the need for a balance between economic growth and ecological protection. One officer borrowed a leaf from Bernardo Mueller (2019):

The conventional method of formulating public policy is enumerating potential world states given the range of options, giving each state a probability, and computing an expected result by weighing the advantages and disadvantages. This method is effective when there is a clear understanding of the options, and risk can be assigned to uncertainty. However, business and public policy frequently operate in complex realms where it is impossible to imagine every scenario that could arise, much less to put a number on likelihoods, costs, and benefits (officer).

Whether the goal is to enhance public services or develop new programs, the system’s public policy structure makes it difficult or impossible to anticipate, evaluate, and control—all essential components of sound policymaking. Attempts to apply the conventional method to such a domain sometimes result in failure or unexpected effects. Furthermore, more data and money or hiring brighter experts are generally insufficient to improve the situation. You can only get so far by improving upon a previously unsuccessful strategy (officer).

I don’t see any advantage in following what I hear from the bush radios about abandoning what works for me and following what I am not confident about unless the advocates give me some incentives (farmer).

## Discussion

Owing to the multifarious nature of policy success [44], this qualitative design exploratory phenomenological investigation aimed to examine the level of awareness about the environmental protection policies among farmers in agricultural production as a significant condition for successful policy implementation. Sustainable farming and prolonged sustainable economic development hinge partly on stakeholders’ knowledge, awareness, and involvement in policy development, implementation, and evaluation.

Inadequate stakeholder coordination in the Ayeyarwady Basin significantly hampers environmental policy implementation. Fragmented efforts among governmental bodies, non-governmental organizations, and communities lead to inefficiency, overlapping initiatives, and policy gaps, resulting in a disjointed approach and reduced effectiveness of environmental measures, as seen in the case in India. Furthermore, the lack of collaboration undermines the formulation of holistic, locally tailored policies essential for comprehensive environmental management. This deficiency also affects resource allocation, leading to suboptimal use of funds, human resources, and technology, and exacerbates conflicts between ecological conservation and developmental objectives. To minimize the chance of such conflicts, Banda et al. emphasize the importance of tailored interventions and support mechanisms addressing challenges within given demographics [45]. Thus, consistent with extant literature, our results indicate that coordinated efforts are crucial for effective environmental policy execution [31, 46–51].

The scarcity of adequate funding critically impairs the execution of environmental protection policies. This financial insufficiency manifests in several vital areas: diminished capacity for enforcement and monitoring of environmental regulations, constrained initiation and sustainability of conservation projects, limited scope in conducting public awareness and educational campaigns and delayed or aborted development of essential ecological infrastructure. Echoing the appeal by extant studies for targeted technical and financial interventions [5, 52–54], restricted funding adversely affects research and development initiatives vital for informed policymaking and curtails training and capacity building necessary for effective policy implementation. It hampers collaborative efforts with various stakeholders in Ayeyarwady. Collectively, these factors contribute to the diminished efficacy of environmental policies in the region. Our findings corroborate extant literature on the role of funding in policy implementation in East Asia and beyond [46, 47, 55, 56].

Farmers’ lack of awareness leads to non-compliance with regulations and unsustainable practices. This gap hinders the adaptation of policies to local agricultural conditions and impedes community involvement in environmental initiatives. Consequently, it forms a significant obstacle to behavioral changes necessary for ecological sustainability. These findings support findings in several previous studies [46–49, 56–62]. Like the Scotland experience [52], the Ayeyarwady farmers observed that policies that contradict their immediate needs were oppressive. The likelihood of a successful conclusion will increase if potential implementation issues are considered during the policy design stage. Cultural, Psychological, and behavioral barriers play a significant role in implementing policy in agriculture in the economic region [55].

Farmers do not understand the theory of cause and effect underpinning the policy. The Myanmar experience with the implementation of public policy is not a unique phenomenon. This finding or result is consistent with the literature advocating heightened stakeholder awareness of ecological policy implementation using advanced technological tools [13, 15, 16, 52, 63, 64]. For example, Kenya took a long time to develop a tobacco control policy, but the one that exists now is in line with international initiatives [65]. The implementation is still lacking, and this may be improved by giving all essential stakeholders the resources they need and keeping them engaged. The authors recommended that policymakers maintain ongoing contact with political leadership and a constant worldwide information exchange [65]. Corroborating extant literature, this study finds that Ayeyarwady farmers resist policy [66–69] [without incentives].

Civil instability impedes policy implementation as the government diverts resources to immediate political and security issues, reducing policy priority, weakening governance capacity, and leading to corruption and mismanagement. Communication disruptions hindered data collection and strained international relations, further complicating policy execution. The erosion of trust and social capital in communities disrupts consensus-building. Such instability results in policy uncertainty and delays, collectively impairing the effective execution of environmental policies. Existing studies also highlight the negative impact of incoherent policy frameworks: lack of a supportive social and political environment impedes policy implementation [70–73].

Similarly, civil unrest causes the stakeholders to work under panic and against time, rendering the output imperfect [55]. Due to perceived production risks, smallholder farmers resist mitigation as they frequently worry about the effects on competitiveness or food security, particularly among impoverished farmers [55]. Occasionally, “conflicting values and interests” result from institutional overlaps between ministries whose responsibilities are unclear, resulting in implementation inconsistencies. As a result of having a distinct ministry for forestry and plantations, Malaysia, for example, promoted the lucrative rubber industry to the detriment of its own forest protection goals [44, 55, 69, 74].

Consequently, “financial capacity” limits the human and operational capacity required for effective climate change governance. According to the literature review, financial capacity emerged as a significant determinant of successful policy design and implementation, indirectly affecting institutional, informational, and technical barriers. A recent report from the UNFCCC indicated that of the 133 developing countries analyzed, economic and financial barriers are the most significant factor in adaptation and mitigation efforts [65].

Political pressure is an additional factor contributing to implementation failures. Political pressure has always been present and complicated during implementation. The efficient operation of bureaucracy is impossible under such pressure. The disconnect between the federal and state governments has negatively impacted implementing policies. The protection of the environment is highly centralized. As a result, health and environmental quality—including the availability of clean water, air, and decent living conditions—may not significantly improve despite the central Government’s enthusiasm for implementing novel population, health, education, and environmental policies and programs [31, 55].

## Conclusion

The study aimed to analyze the level of awareness among farmers about environmental protection policy concerning agricultural productivity as a critical factor in vulnerable Ayeyarwady Basin economic development. The study employed a qualitative design. It was a phenomenological study using the interpretivism technique.

Much as Myanmar has made tremendous strides towards ensuring biodiversity and ecological conservation through public policy that is consistent with the conventions of the United Nations Sustainable Development, according to the results, there is a significant difference between the perceptions and experiences of the farmers on the one hand, and those of the public servants and academia on the other. The academia and the public officers seem to have a sound background knowledge of the policy, rules, and regulations to safeguard ecological treasures. They also indicate a profound understanding of the importance of such a policy framework in ensuring sustainable agrarian activities and bolstering sustainable economic development in Myanmar.

However, it is now recognized that the policy context is far more complex than before [51, 75]. The government must realize that more work needs to be done to attempt to guarantee that goals are translated into results or, to put it another way, that policy failure is avoided as understanding has grown. Governments must show interest in measures to assist and strengthen the policy process, particularly during the implementation phase, rather than letting it fall into complete or partial failure [51].

The study identified a differential in policy awareness among farmers: a segment lacked awareness, while others, although recognizing the policies, admitted to limited understanding. This variation in awareness was linked to diverse factors, including the farmers’ literacy and geographic positioning. These findings underscore the necessity for enhanced awareness initiatives, advocacy for regulations, and more collaborative policy development approaches. Such efforts are crucial for the effective implementation of the United Nations’ Sustainable Development Goals by 2030 in Myanmar, indicating that significant progress is still required for the successful realization of these policy objectives. The relevance of this study is highlighted by its unique evaluation of environmental regulation awareness among farmers in the Ayeyarwady River Basin. It provides crucial insights into the relationship between ecological regulations and economic development, particularly in global climate change. These insights are vital for formulating policies that balance economic resilience, ecosystem protection, and sustainable growth. Banda et al. advocate for compassionate and contextually sensitive interventions, a recommendation that is equally pertinent for addressing the long-term impacts of policies [45]. Moreover, the study contributes to the broader discourse on Sustainable Development Goals by amalgamating diverse perspectives from farmers, public officials, and experts. This inclusive approach is essential in guiding policy decisions that align agricultural economic growth with biodiversity conservation, thereby influencing the development of a more sustainable and equitable future in Myanmar.

### Recommendations

The study advocates for a strategic, multi-layered approach to environmental policy implementation, affecting agricultural systems and broader economic levels. Consistent with Uddin et al. [5], emphasizing collaborative efforts, especially in Myanmar, it suggests extensive awareness campaigns by ministries responsible for Natural Resources, Agriculture, and Information to educate farmers about environmental conservation and its relevance to agriculture.

A dual strategy is proposed to engage farmers, merging traditional outreach and modern media, enhancing their understanding of sustainability and its agricultural and ecological implications. The study also stresses the importance of farmers protecting critical resources like land, water, and air through research-based policies, underscoring the interconnectedness of agriculture and environmental health.

It highlights the need for effective administrative enforcement and strategic environmental protection planning, including innovative solutions to operational challenges, to enhance environmental quality. We noted officers’ reluctance to critique policies was probably attributable to several interconnected factors: a strong sense of organizational loyalty leading to a hesitation to express criticism, fear of professional repercussions potentially impacting credibility, cognitive dissonance causing a conflict between self-perception and acknowledging policy failures, and concern over personal accountability. Additionally, there is a tendency to protect the organization’s public image, a limited perspective on the policy’s broader impact leading to biased assessments, and an optimism bias, especially among those deeply invested in the policy’s implementation, skewing their perception of its success and failures. These factors collectively create a challenging environment for officers to evaluate candid policy. More prompts and continuous assurance about anonymity and confidentiality helped the situation.

There was a variability in participant adherence to prearranged interview schedules. This led to overstretching the budget and timelines. This necessitated the adoption of more flexible scheduling approaches to accommodate the diverse availability of the participants.

The study suggests a subsequent investigation into the effects of advanced communication strategies and infrastructure on implementing environmental policies among Ayeyarwady River Basin farmers. This proposed research would evaluate the efficacy of various communication techniques and infrastructures in augmenting farmers’ comprehension and awareness of these policies. Additionally, it would analyze the relationship between enhanced communication and actual policy compliance, offering insights into the impact of policy awareness on environmental practices within agricultural contexts.

## Data Availability

Raw data may be available through the corresponding author, who is also the submitting author.
